# Acute Abdomen and Perforated Bowel with a Rare Pathology: Nonfamilial Visceral Myopathy

**DOI:** 10.1155/2011/645349

**Published:** 2011-12-08

**Authors:** Jakob Burcharth, Caroline Olsen, Jacob Rosenberg

**Affiliations:** ^1^Department of Surgical Gastroenterology, Herlev Hospital, University of Copenhagen, Herlev Ringvej 75, 2730 Herlev, Denmark; ^2^Department of Pathology, Herlev Hospital, University of Copenhagen, Herlev Ringvej 75, 2730 Herlev, Denmark

## Abstract

Visceral myopathy is a rare chronic disease affecting the peristalsis of the bowel causing intermittent pseudoobstruction. We report an atypical case of an eighty-nine-year-old woman with no prior history of abdominal illness who was admitted to our hospital with 2 days of increasing nausea, abdominal distension, and abdominal pain. On arrival at the hospital, she was critically ill. Abdominal X-ray showed distended loops of the colon and liquid levels resembling colonic obstruction. A subsequent abdominal CT scan confirmed the colonic obstruction. A suspicion of sigmoid volvulus was raised, that is why a barium enema was performed but no lower colonic obstruction could be confirmed. Acute laparotomy showed perforated cecum without intestinal obstruction. Postoperatively, the patient became septic which was fatal for the patient. Pathology gave the diagnosis visceral myopathy. It is very difficult to make the diagnosis clinically and radiologically since visceral myopathy mimics other more common gastrointestinal diseases. It is important to consider visceral myopathy as a possible diagnosis in cases with recurrent episodes of abdominal pain, vomiting, and abdominal distension, but without actual intestinal obstruction.

## 1. Introduction

Visceral myopathy (VM) is a rare, severe, and often misdiagnosed pathological condition. Patients with VM suffer from recurrent disturbances of motility in the gastrointestinal tract which leads to failure in propulsive peristalsis and intermittent pseudoobstruction [1]. The normal clinical picture of a patient with VM is characterized by a chronic course dominated by recurrent episodes of abdominal pain, vomiting, and abdominal distension. Important diagnostic criteria include absence of mechanical obstruction, and histological examination of all intestinal layers [2]. The diagnosis can be delayed due to the rarity of VM, great variation in symptoms, and the many similarities VM has with other more common gastrointestinal diseases. Furthermore, there is almost always absence of specific radiological features that indicates the diagnosis VM [2]. We report a rare and atypical case of VM with acute bowel perforation and a fatal outcome for the patient.

## 2. Case Report

**Figure 1:**
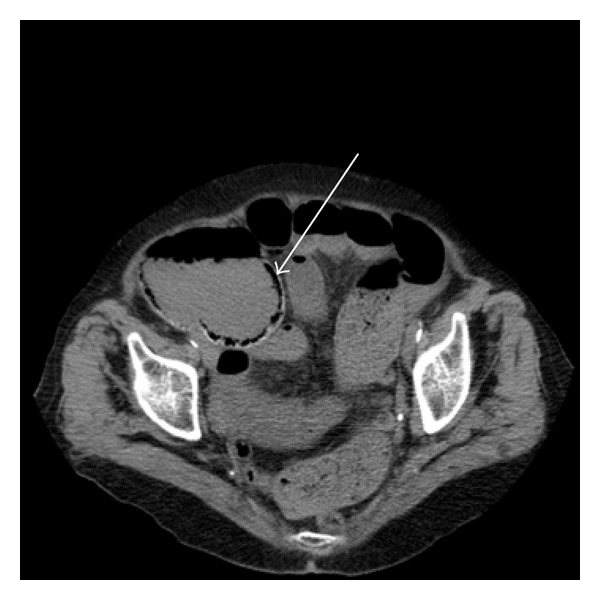
Abdominal CT scan showing air entrapment in the right colonic wall (marked by arrow), colonic liquid levels resembling colonic obstruction, and suspicion of sigmoidal volvulus.

**Figure 2:**
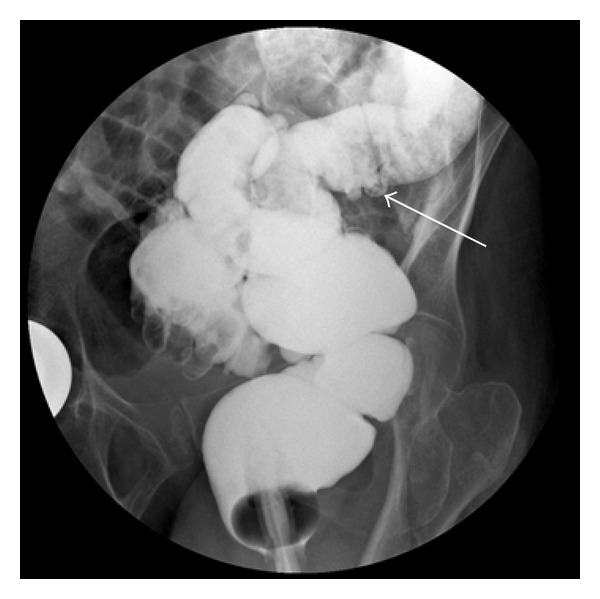
Barium enema showing diverticulosis (marked by arrow) but no signs of sigmoidal obstruction.

An eighty-nine-year-old woman was admitted to our hospital with 2 days of abdominal pain, nausea, vomiting, and fatigue. In the last 5 days, she had symptoms of constipation. She had no history of earlier abdominal diseases or symptoms. Abdominal plain X-ray in the upright position showed distended loops of the colon and liquid levels resembling colonic obstruction. An abdominal CT scan was performed which confirmed colonic obstruction and possible air entrapment in the right colonic wall which was interpreted as caused by sigmoid volvulus (Figure 1). A subsequent barium enema could not confirm sigmoid volvulus (Figure 2) or any other kind of lower colonic obstruction. The laboratory test results showed leukocytes 8.4 bn/litre, glomerular filtration rate >60 mL/min, blood haemoglobin 7.5 mmol/litre, plasma c-reactive protein 33 mg/litre, plasma potassium 3.3 mmol/litre, and plasma sodium 140 mmol/litre. The patient was stabilized with intravenous liquids, and an operation was scheduled acutely due to the patient's critical condition. Intraoperatively, a bowel perforation in the caecum was found. Colon was constipated to a minor extent. Faecal peritoneal soilage was found, and a right-sided hemicolectomy was performed with a terminal ileostomy. A search was made for an intraabdominal tumour but none was found. During the procedure, the patient received broad-spectrum antibiotics. Postoperatively, the patient developed hypotension most likely due to sepsis. Despite intravenous fluid administration, antibiotics, and dopamine infusion, the patient did not improve. The patient had no wish of receiving intensive care and died the following day without attempts of resuscitation as per the patient's request.

## 3. Pathology Results

**Figure 3:**
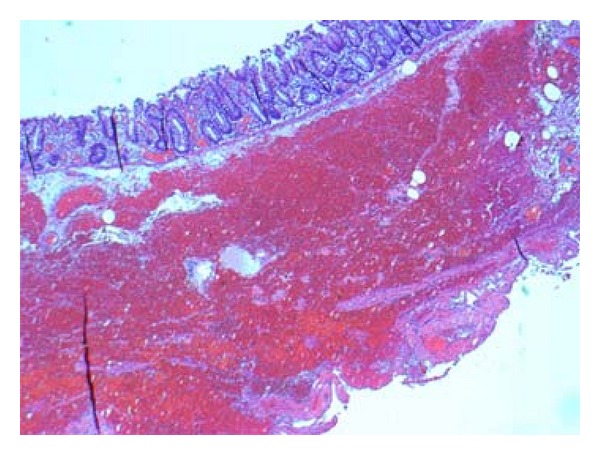
Intestinal biopsy showing clear signs of myopathy and a degenerated muscular layer.

The pathology examination was based on full-thickness biopsies obtained from the operative specimen. Tissue sections were cut into 3-4 microns and dyed with haematoxylin-eosin. In distended areas, the bowel wall was only 1 mm thick but the mucosa was intact with normal thickness and was fully vital with no signs of ischaemia. The submucosa showed hyperaemia and expansive bleeding, but no fibrosis. The inner and outer muscular layers were equally affected by necrosis showing vacuolar degeneration and abruption of muscle fibres together with bleeding, neutrophilic inflammation, and fibrosis. In some areas, the muscle layer was completely absent. The nerve ganglia were normal in number and morphology. The conclusion was nonfamilial visceral myopathy (Figure 3).

## 4. Discussion

VM is part of a heterogeneous group of diseases called intestinal pseudoobstruction (IP). This rare group of diseases can be inherited (familial visceral myopathy), primary idiopathic (nonfamilial visceral myopathy), or secondary to other diseases (scleroderma, systemic lupus, amyloidosis, Ehlers-Danlos syndrome, stroke, or encephalitis) [3–5]. Patients suffering from VM vary greatly in their clinical manifestations. Some patients are completely asymptomatic, some patients will experience light abdominal symptoms such as nausea, vomiting, postprandial pain, abdominal distension, and diarrhea, and few patients experience repetitive attacks of bowel obstruction resembling small bowel or colonic obstruction [3]. The patients are often malnourished from the decreased intestinal motility [1].

Treatment of VM consists of fluid and electrolytes by IV infusions and gastric and colonic decompression by nasogastric and rectal tubes. Pitt et al. [6] showed that total parenteral nutrition (TPN) and ventral enterostomy greatly reduced the number of required admissions in a group of patients with chronic intestinal pseudoobstruction. Surgical resections and enterostomies is an option in patients with localized involvement of the gastrointestinal tract who do not respond to conservative treatment [7]. Surgical resection as treatment of nonlocalized VM often have disappointing results, since bowel affection will expand further than the resected specimen [1]. The surgical consensus is to avoid exploratory surgery except when evidence of mechanical obstruction or intestinal perforation exists [8]. In this case, the patients colon was constipated to a minor extent, yet not enough to explain the caecal perforation. We therefore suppose that the caecum perforation resulted from VM rather than constipation. The surgeon chose to perform a right-sided hemicolectomy with a permanent terminal ileostomy. This procedure was considered the best option for this patient, but a damage control caecostomy could also be considered in a critically ill patient with intestinal perforation.

The present case is clinically interesting and very exceptional because of several factors: the fast deterioration of the patient's clinical condition, the total absence of prior abdominal symptoms, and the late age of debut. To our knowledge, there are no reported cases of VM that debut with bowel perforation combined with no prior abdominal symptoms. The vast majority of the literature describes VM as a latent chronic disease with occasional relapsing attacks of pseudoobstruction and with slow progression of intestinal failure. Bowel perforation without any prior symptoms is a very atypical presentation of VM and is normally considered a differential diagnosis which should be excluded [9].

VM is a very rare group of diseases, and they should be considered whenever a patient presents with uncharacteristic abdominal symptoms, recurrent attacks of abdominal distension, and pain with no radiological evidence of intestinal obstruction. This case, however, also shows that VM in extremely rare cases can present with sudden debut of acute abdomen and no prior abdominal symptoms.

## 5. Learning Points

- The symptoms of visceral myopathy are unspecific and often mimic other more common gastrointestinal diseases.
- In patients that present with recurrent episodes of abdominal distension with no evidence of mechanical obstruction, VM should be considered.
- It is important to consider VM no matter the patients age.
- Perforated bowel due to VM is very rare but should be considered in clinically relevant cases.
